# Real-world data in primary care: validation of diagnosis of atrial fibrillation in primary care electronic medical records and estimated prevalence among consulting patients’

**DOI:** 10.1186/s12875-022-01961-y

**Published:** 2023-01-04

**Authors:** C. de Burgos-Lunar, I. del Cura-González, J. Cárdenas-Valladolid, P. Gómez-Campelo, J. C. Abánades-Herranz, A. López-de Andrés, M. Sotos-Prieto, V. Iriarte-Campo, M. A. Salinero-Fort

**Affiliations:** 1 Department of Preventive Medicine, Hospital Universitario Clínico de San Carlos, Madrid, Spain; 2 Research Network on Chronicity, Primary Care and Health Promotion -RICAPPS-(RICORS), Madrid, Spain; 3 Research Unit, Primary Health Care Management, Madrid, Spain; 4 Department of Medical Specialties and Public Health, Faculty of Health Sciences Rey Juan Carlos University, Madrid, Spain; 5 Information Systems Department, Primary Health Care Management, Madrid, Spain; 6 Biosanitary Research and Innovation Foundation of Primary Care (FIIBAP), Madrid, Spain; 7 The Hospital La Paz Institute for Health Research (IdiPAZ), Madrid, Spain; 8 Alfonso X El Sabio University, Madrid, Spain; 9 Monovar Health Center, Madrid, Spain; 10 Department of Public Health & Maternal and Child Health, Faculty of Medicine, Universidad Complutense de Madrid, Madrid, Spain; 11 Department of Preventive Medicine and Public health, Universidad Autónoma de Madrid, Madrid, Spain; 12 CIBERESP (CIBER of Epidemiology and PublicHealth), Madrid, Spain; 13 Department of Environmental Health, Harvard T.H.Chan School of Public Health, Boston, MA USA; 14 General Subdirectorate of Research and Documentation, Department of Health, Madrid, Spain

**Keywords:** Atrial fibrillation, Prevalence, Electronic health records, Validation

## Abstract

**Background:**

Primary care electronic medical records contain clinical-administrative information on a high percentage of the population. Before this information can be used for epidemiological purposes, its quality must be verified.

This study aims to validate diagnoses of atrial fibrillation (AF) recorded in primary care electronic medical records and to estimate the prevalence of AF in the population attending primary care consultations.

**Methods:**

We performed a cross-sectional validation study of all diagnoses of AF recorded in primary care electronic medical records in Madrid (Spain).

We also performed simple random sampling of diagnoses of AF (ICPC-2 code K78) registered by 55 physicians and random age- and sex-matched sampling of the records that included a diagnosis of AF. Electrocardiograms, echocardiograms, and hospital discharge or cardiology clinic reports were matched.

Sensitivity, specificity, positive and negative predictive values (PPV and NPV), and overall agreement were calculated using the kappa statistic (κ). The prevalence of AF in the community of Madrid was estimated considering the sensitivity and specificity obtained in the validation. All calculations were performed overall and by sex and age groups.

**Results:**

The degree of agreement was very high (κ = 0.952), with a sensitivity of 97.84%, specificity of 97.39%, PPV of 97.37%, and NPV of 97.85%.

The prevalence of AF in the population aged over 18 years was 2.41% (95%CI 2.39–2.42% [2.25% in women and 2.58% in men]). This increased progressively with age, reaching 16.95% in those over 80 years of age (15.5% in women and 19.44% in men).

**Conclusions:**

The validation results obtained enable diagnosis of AF recorded in primary care to be used as a tool for epidemiological studies.

A high prevalence of AF was found, especially in older patients.

## Introduction

Clinical databases are increasingly used as sources of information for research. They facilitate the availability of large samples and reduce the time and resources needed to obtain results.

Several studies have successfully evaluated the quality and completeness of the information contained in clinical-administrative databases. While some of these studies have been carried out in the primary care setting [1–3], most have been conducted in the hospital or specialist practice setting using ICD-9 or ICD-10 coding to select patients [4–8].

In order to perform prevalence studies and maximize the use of risk estimation models, the population studied must be comparable to the population to which the results will be extrapolated. Consequently, information is best sourced from primary care.

In the Spanish National Health System, primary care offers universal coverage and continuous free access for the entire population, and the general practitioner (GP) is the gatekeeper to visit medical specialists. The flow of information between primary care and medical specialists is usually necessary for continuity of care, prescription of medication (partially or fully financed), and obtaining support for therapeutic adherence. Patient data are recorded using electronic medical records (EMR), and the specialists’ diagnoses are accessible through the HORUS viewer in the primary care EMR.

Patient data are recorded using electronic medical records (EMR). Furthermore, primary care EMR designed for care purposes enable epidemiological research to be carried out based on real-world data [9–11]. To guarantee the validity of this information for research purposes, it is necessary to evaluate its quality and completeness.

Although there has not always been excellent accuracy in validating primary care electronic medical records [12], specific diagnoses such as atrial fibrillation have been validated with good results in Canada [13] using the International Classification of Disease, 9th Revision.

This study aims to validate diagnoses of atrial fibrillation (AF) in primary care EMR and to estimate the prevalence of this disease in people over 18 years attending primary care consultations in Madrid (Spain).

## Methods

### Design

Cross-sectional study, to validate the diagnosis of AF in primary care EMR.

### Data source

Fifty-five family doctors participated in the validation study in 43 health centres in Madrid (Spain).

The prevalence study was carried out with information from the EMR of all the health centres in Madrid (262 centres).

### Sources of information

The information was based on individualized data from patients’ primary care EMR. All health centres in Madrid have had EMRs for more than 15 years. The primary care EMR from the AP-Madrid database is structured around a list of episodes consisting of a code and a description of the diagnosis or name. The code corresponds to the second edition of the International Classification of Primary Care (ICPC-2) and can have several descriptions [14, 15]. Furthermore, these codes sometimes need to be later amended or changed entirely. In that case, it can be done in two ways: replacing the code with another (deleting the previous one and selecting the new code) or deleting the diagnosis description and writing the new one. The second procedure is faster; some professionals do it when they have little time available for consultation. For this reason, in the validation procedure of atrial fibrillation, it is necessary to include records whose description refers to the atrial fibrillation but not the ICPC-2 code.

The professionals have an ICPC-2 code selection assistant that allows you to search and register by literal/descriptor of the reason for the pathology of the patient. The physician already has training in this issue through a 20-hour course. Then, the software application assigns the code corresponding to the selected descriptor. In addition, both the code and the descriptor are visible to the professional in the ICPC-2 selection assistant, which has led, over the years, to some professionals learning and selecting the event through the code, as it is a more efficient procedure. For AF, the ICPF-2 code is “K78”, with the diagnostic label “atrial fibrillation / atrial flutter,” which corresponds to ICD-9 code “427.31” and ICD-10 code “I48”.

### Study population

The study population comprised persons aged 18 years or older with a primary care EMR from the AP-Madrid database who had at least one record before 1 January 2015. Patients temporarily displaced from their usual place of residence and patients who had a temporary assignment due to demand for specific medical care were excluded.

### Samples

A previous systematic review has analyzed studies of validating diagnosis of atrial fibrillation [16], but we have not found any in primary care patients older than 18. Therefore, given the absence of reference information on the proportion of incorrectly classified cases (false negatives and false positives), maximum indetermination was assumed (p = q = 0.5). With this assumption, and to obtain a confidence interval (CI) of 95% and a precision of 5%, the required sample size was 384 patients. We increased this up to 423 to adjust for a foreseeable loss of 10% between sampling and validation of the diagnosis (change of address, death, and other reasons).

Two patient samples were obtained:

- Sample 1: 423 patients with an AF code (CIAP-2 K78). This was obtained by simple random sampling from the list of patients of the participating GP.
- Sample 2: 423 patients without an AF code. Given that the probability of presenting AF increases with age and its distribution is not equally prevalent in both sexes, sample 2 was matched with sample 1 for the variables year of birth and sex. This approach aimed to avoid overestimation of specificity if the sample is represented by the less prevalent age and sex strata.

### General practitioners

Our group usually collaborates with 153 GP who work in health centers representative of the seven health areas of community of Madrid (urban and rural centers, with and without family residents, low and medium-high income). From these, 55 were selected by random sampling. Their participation in the project was encouraged with a certificate of participation that allowed them to prove their research activity and comply with the Madrid health services agreement. The average number of AF cases per professional was 30.

### Inclusion criteria and protocol

Subjects were considered to have AF if they met any of the following criteria:

- Irregular rhythm on the electrocardiogram, with no P wave, but instead rapid fibrillatory waves of different shapes, sizes, and rhythms, leading to an irregular ventricular response.
- Absence of a wave of the mitral valve movement in the echocardiogram.
- An ICD code diagnosis of AF (ICD9 code “427.31” or ICD10 code “I48”) in the hospital discharge report or cardiology outpatient report.

**Fig. 1:**
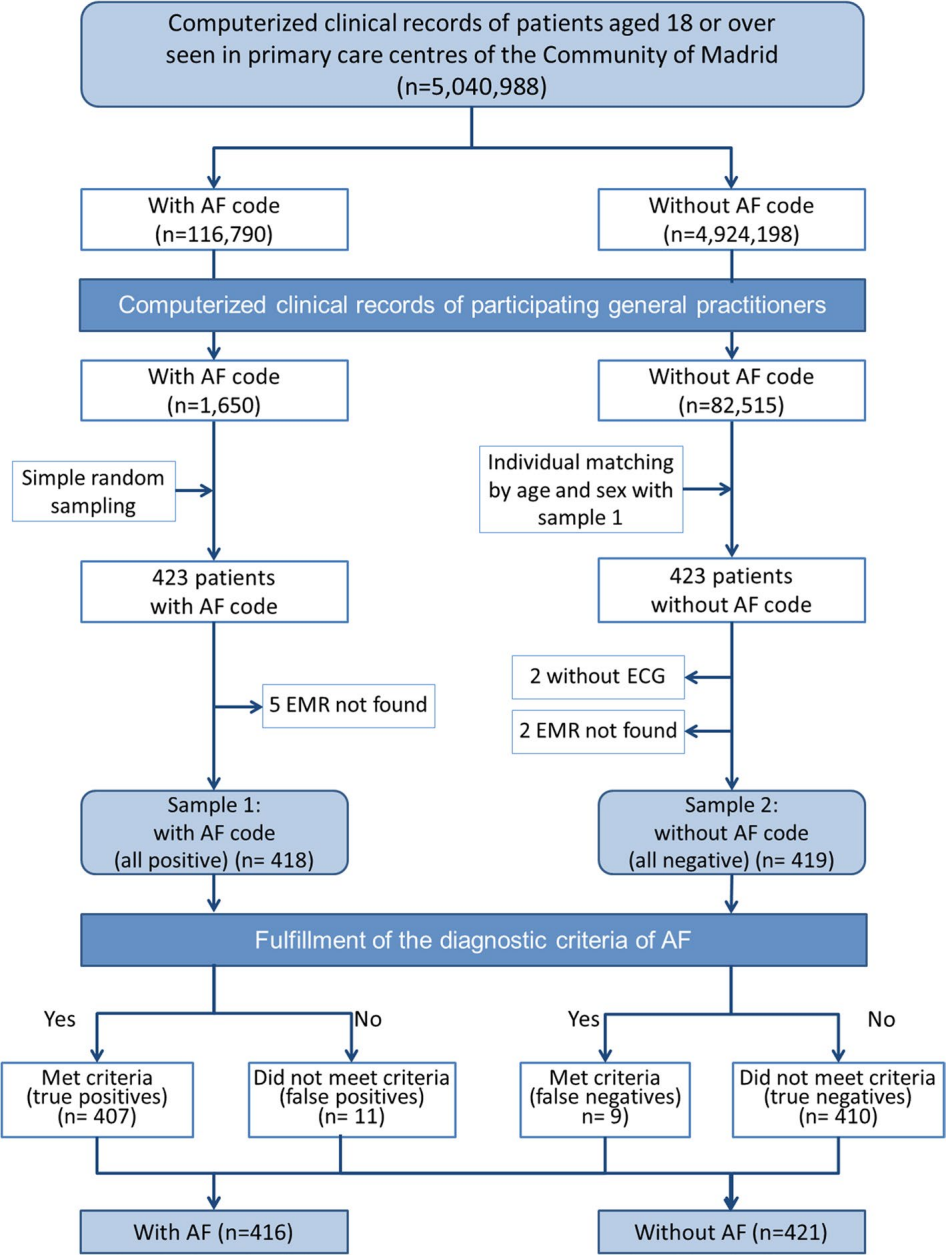
Validation algorithm flowchart

To validate the diagnoses, the evaluators accessed the patients’ primary healthcare EMR and, based on the information shown, verified compliance with the criteria. The validation algorithm is shown in Fig. 1.

The evaluators were 12 GP with experience in the management of the AP-Madrid database who had previously received appropriate training. Furthermore, all had completed the specialty of family and community medicine, which in Spain has a duration of four years and in which they remain for one year rotating in internal medicine and two months in cardiology, so their familiarization with FA diagnosis and the gold standard was assured. Also, the evaluation was peer-reviewed, and discrepancies were resolved by consensus.

### Statistical analysis

First, a descriptive analysis of the study populations and samples was performed. Age was expressed as the median and interquartile range (IQR), and qualitative variables were summarized with their relative frequency.

Sensitivity, specificity, positive predictive value (PPV), and negative predictive value (NPV) were calculated with their 95% confidence intervals (CI) overall and by sex and age groups. We tested whether the sensitivity and specificity differed according to the different categories of the variables using the χ^2^ test of homogeneity between proportions. When the conditions for application were not met (any expected frequency less than five), a two-sided Fisher’s exact test was used.

Sensitivity is the proportion of cases with AF codes on the primary care EMR among all cases that could be verified as meeting the diagnostic criteria (true positive). Specificity is the proportion of cases without AF codes in the primary care EMR among cases that did not meet the diagnostic criteria (true negative).

The proportion of individuals with a disease code in the primary care EMR (apparent prevalence) should not be used to estimate the prevalence of a disease in that population, because the sensitivity and specificity of these diagnoses are usually less than 100%. Therefore, since the proportion of individuals with a positive result includes false positives and excludes false negatives, estimating the true prevalence of a disease requires adjustment for misclassification resulting from the sensitivity and specificity. In this study, the formula used was that proposed by Rogan and Gladen for this adjustment [17]. True prevalence was calculated as [apparent prevalence + specificity – 1] / [sensitivity + specificity – 1].

The degree of overall agreement between the recorded diagnosis and the reference standard and interobserver agreement was determined using the kappa index (κ) and its CIs. According to this value, agreement was considered as poor (≤ 0.20), low (0.21–0.40), moderate (0.41–0.60), good (0.61–0.80), or very good (≥ 0.81) [18].

Data were analyzed using SPSS for Windows, V.19.0 (IBM Corp., Armonk, New York, USA). The CI of the κ index and the predicted values were calculated using the macros for SPSS from the Laboratorio de Estadística Aplicada, Universidad Autónoma de Barcelona (!KAPPA and!DT, respectively) [19, 20].

## Results

The main demographic characteristics of the study population seen in Madrid with episodes of AF (ICPC-2 K78) recorded in primary care EMR and those of the selected samples are shown in Table 1.

**Table 1:** Main demographic characteristics of the population of patients aged over 18 years in primary care centers and of sample

	N (%)	Age (years) median (IQR)	Age ≥ 70 years (%)	Female sex (%)
**Patients with diagnostic code for atrial fibrilation (CIAP K-78)**	116,790 (2.32)	79.04 (70.32–85.34)	75.76	49.55
Sample of patients with diagnosis code of atrial fibrillation (sample 1)	418 (0.36)	78.69 (70.94–84.73)	77.75	49.76
− Correct diagnosis (TP)	407 (97.37)	78.96 (71.09–84.77)	78.62	50.37
− Incorrect diagnosis (FP)	11 (2.63)	69.97 (45.07–74.06)	45.45	27.27
**Patients without diagnostic code for atrial fibrillation (K-78)**	4,924,198 (97.68)	47.18 (36.48–60.87)	13.89	52.72
Sample of patients without diagnosis code of atrial fibrillation (sample 2)	419 (0.01)	78.40 (70.87–84.66)	77.09	49.16
− Correct diagnosis (TN)	410 (97.85)	77.37 (70.83–84.57)	76.83	49.02
− Incorrect diagnosis (FN)	9 (2.15)	85.38 (81.37–88.52)	55.56	88.89

*IQR* interquartile range, *TP* true positive, *FP* false positive, *TN* true negative, *FN* false negative

Males with an active primary care EMR who were regular users of the health centres accounted for 47.36% of the 5,040,988 patients over 18 years of age. The mean age of the study population was 49.82 (SD 17.74) years.

The analysis revealed that 2.32% of the population had an AF code in their primary care EMR, with a mean age of 76.87 (SD 11.76) years. Of these, 50.45% were male. Males accounted for 50.24% of the patients included in sample 1 (with an AF code), and their mean age was 77.08 (SD 10.9) years.

As shown in Table 2, the diagnosis of AF was confirmed in 97.84% of cases (sensitivity), with no significant differences when patients were stratified by age group or sex.

**Table 2:** Predictive values, sensitivity, specificity, and agreement for a diagnosis of atrial fibrillation

	TP	FP	FN	TN	PPV (95% CI)	NPV (95% CI)	Sensitivity % (95% CI)	χ^**2**^***p***-value	Specificity % (95% CI)	χ^**2**^***p***-value	Diagnostic agreement kappa (95% CI)
**Overall**	407	11	9	410	97.37 (95.35–98.52)	97.85 (95.97–98.87)	97.84 (95.97–98.87)		97.39 (95.35–98.52)		0.952 (0.932–0.973)
Female	205	3	5	201	98.56 (95.85–99.51)	97.57 (94.45–98.96)	97.62 (94.45–98.96)	0.630^a^	98.53 (95.85–99.51)	0.018^a^	0.961 (0.935–0.988)
Male	202	8	4	209	96.19 (92.66–98.06)	98.12 (95.27–99.27)	98.06 (95.27–99.27)		96.31 (92.66–98.06)		0.943 (0.912–0.975)
Age < 70	87	6	1	95	93.55 (86.63–97.01)	98.96 (94.33–99.82)	98.86 (94.33–99.82)	0.467^a^	94.06 (86.63–97.01)	0.146^a^	0.926 (0.872–0.980)
Age ≥ 70	320	5	8	315	98.46 (96.45–99.34)	97.52 (95.19–98.74)	97.56 (95.19–98.74)		98.44 (96.46–99.34)		0.960 (0.938–0.981)

^a^Fisher’s exact test (two-sided); *TP* true positive, *FP* false positive, *FN* false negative, *TN* true negative, *PPV* positive predictive value, *NPV* negative predictive value, *95% CI* 95% confidence interval

In seven of the 11 cases where the diagnosis could not be confirmed, the general practitioners had changed the title of the diagnosis without changing the code (two AV blocks, one left atrial rhabdomyoma, one ventricular arrhythmia, one patent foramen ovale, one lower limb arterial thrombosis, and one anxiety episode). In four cases, no electrocardiograms, or hospital or cardiology reports were found to support the diagnosis.

The criteria for AF were not met by 97.39% of those who had no recorded diagnosis of AF (specificity), with significantly lower proportion in men than women (96.31% vs. 98.53%; *p* = 0.018).

In four of the nine cases that presented AF but did not have the code recorded, the general practitioners changed the diagnostic label without modifying the K78 ICPC-2 code (three tachycardias and one ventricular arrhythmia). In a further three cases, hospital reports were found with a diagnosis of AF, two of them within the previous month. Two additional cases had a diagnosis of AF included within another episode (stroke).

The overall degree of agreement between the diagnosis recorded in the primary care EMR and the reference standard, measured as the κ index, was very good (κ = 0.952), both overall and for the different strata of the variables sex and age over 69 years. κ indices above 0.900 were achieved in all cases.

The overall agreement between observers was also very good (κ = 0.862).

The true prevalence of AF in individuals over 18 years of age was 2.41% (2.25% in women and 2.58% in men). However, this increased progressively with age, reaching 16.95% in those over 80 years of age (15.5% in women and 19.44% in men).

Table 3 shows the differences in the prevalence of AF, as recorded in the primary care EMR (apparent prevalence) and according to the reference standard (true prevalence), both overall and by age group and sex.

**Table 3:** Apparent prevalence (diagnosis in the EMR) and true prevalence (fulfilment of the diagnostic criteria) of AF in patients aged 18 or over in primary health care centers of the Community of Madrid

**ALL**	**N**	**n**	**Apparent prevalence**	**True prevalence**
**Total**	**5,040,988**	**116,790**	**2.32 (2.3–2.33)**	**2.41 (2.39–2.42)**
18–50	2,761,166	3578	0.13 (0.13–0.13)	0.11 (0.1–0.11)
>50–60	880,580	6826	0.78 (0.76–0.79)	0.79 (0.77–0.81)
>60–70	626,926	17,901	2.86 (2.81–2.9)	2.97 (2.92–3.01)
>70–80	436,045	34,110	7.82 (7.74–7.9)	8.19 (8.11–8.27)
>80	336,271	54,375	16.17 (16.05–16.29)	16.95 (16.95–17.08)
**FEMALE**	**N**	**n**	**Apparent prevalence**	**True prevalence**
**Total**	**2,653,677**	**57,871**	**2.18 (2.16–2.2)**	**2.25 (2.24–2.27)**
18–50	1,395,907	778	0.06 (0.05–0.06)	0.04 (0.04–0.05)
>50–60	456,252	1961	0.43 (0.41–0.45)	0.43 (0.41–0.42)
>60–70	338,843	6785	2 (1.96–2.05)	2.07 (2.02–2.12)
>70–80	244,789	15,739	6.43 (6.33–6.53)	6.67 (6.57–6.77)
>80	217,886	32,608	14.97 (14.82–15.12)	15.55 (15.4–15.7)
**MALE**	**N**	**n**	**Apparent prevalence**	**True prevalence**
**Total**	**2,387,311**	**58,919**	**2.47 (2.45–2.49)**	**2.58 (2.56–2.6)**
18–50	1,365,259	2800	0.21 (0.21–0.21)	0.18 (0.17–0.19)
>50–60	424,328	4865	1.15 (1.11–1.18)	1.18 (1.14–1.21)
>60–70	288,083	11,116	3.86 (3.79–3.93)	4.05 (3.98–4.12)
>70–80	191,256	18,371	9.61 (9.47–9.74)	10.14 (10–10.28)
>80	118,385	21,767	18.39 (18.17–18.61)	19.44 (19.22–19.67)

## Discussion

The results of the validation study show very good agreement with the reference standard (κ = 0.952), as well as a high sensitivity (97.84%) and specificity (97.39%) overall and in each sex category and age group for a diagnosis of AF recorded in the primary care EMR.

Other studies evaluating medical databases to identify AF patients have also obtained similar results. For example, in a systematic review of 16 studies, 63% conducted in the hospital setting, PPV ranged from 70 to 96% (median 89%), and sensitivity ranged from 57 to 95% (median 79%). PPV was only reported in three studies and ranged from 97 to 99%. A single study estimated NPV at 98.6% [16].

Most of the studies found validated the ICD-9 code by reviewing medical records. In Canada, Tu et al. [21] incorporated the use of antiarrhythmic drugs, anticoagulants, and cardioversion into the algorithm for selecting AF patients. In Switzerland, Norberg et al. [22] identified cases using ICD-10 codes (PPV, 96.5%), an electronic database of electrocardiographic records (PPV, 88.7%), or both (PPV, 98.1%).

Several authors have considered AF an epidemic disease due to its high prevalence and increasing incidence [23, 24]. Our results show that the prevalence of AF increases with age and is higher in men than in women, in agreement with other publications [25–31].

Prevalence studies show significant variability in the ages considered, the methodology used, the scope of the study, and the types of AF included.

In 2004, the Framingham study found the prevalence of AF to be 0.4–1% in the general population (8% in those over 80 years of age) [25], and in 2001, the ATRIA study, which was performed in the USA, estimated an overall prevalence of 0.95%, rising to 9% in those over 80 years of age [26].

In Europe, the Rotterdam study (2006) found an overall prevalence of 5.5% in persons aged ≥55 years, rising to 15.4% in those aged 80 years or older [27]. In Portugal, the FAMA study (2010) estimated the overall prevalence in the over-40s at 2.5%, increasing to 7.5% in the over-80s [28]. In 2013, Norberg et al. obtained an overall prevalence of 3 and 21.9% in patients over-85 years of age in Sweden [22], and in 2007, Murphy et al. found prevalence values of 0.87 and 7.1% in the over-85 s in Scotland [29].

In Spain, the CARDIOTENS study (1999) estimated an overall prevalence of AF of 4.8% (2.8% in primary care and 17.6% in specialized care). This reached 11.1% in those over 80 years of age (8.3% in primary care and 26.3% in specialized care) [32]. In the PREV-ICTUS study (2007) on the population over 60 years of age attending primary care centres and specialized care consultations, the prevalence of AF in those over 85 years of age was 16.5% [33]. The ESFINGE study (2012) found a prevalence of AF of 31.3% in hospitalized patients over 70 years of age [34]. In addition, the Val-FAAP study (2012) [35], which was conducted in primary care, estimated an overall prevalence of 6.1 and 17.6% in those over 80 years of age. The OFRECE study, which analyzed 8400 patients over 40 years of age seen in primary care, found the prevalence of AF to be 4.9% (4.4% known and 0.5% not known) [30].

**Fig. 2:**
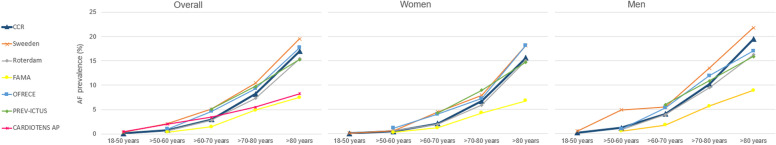
Prevalence of Atrial Fibrillation. Comparisons with other studies

Figure 2 shows the comparison of our results with those of other validation studies.

Prevalence values vary widely depending on the setting where the studies are conducted [32]. Primary care EMR provide information from the entire population attended by all professionals involved in the health-disease process (nurses, medical staff, post-graduate medical residents), thus reducing selection bias, given that the pattern of patient care may differ between participating and non-participating physicians. Similarly, the characteristics of participating patients may differ from those who do not participate.

Our study is subject to a series of limitations. First, the false positives cannot always be considered diagnostic errors, although it was impossible to confirm that the reference criteria were met. This circumstance may have led the prevalence of AF to be underestimated.

Second, the information included may not be exhaustive, as it does not include information on patients treated by a specialist doctor (cardiologist, internist) or private medicine. However, as previously stated in the introduction, the Spanish National Health System provides coverage to 99.1% of the population [36] and double public-private monitoring with shared information is frequent. Primary care is usually the gateway to the health system, where around 90% of health problems are treated and resolved, and it is also where most patients who have been treated at other levels of care return [37, 38]. The Spanish National Health System partially or fully finances medicines prescribed by the public health system, and chronically ill patients usually seek prescriptions. The flow of information between primary care and medical specialists is usually necessary for the continuity of care, and for obtaining support for therapeutic adherence. Patient data are recorded using EMR, and the specialists’ diagnoses are accessible through the HORUS viewer in the primary care EMR. Therefore, we consider that the proportion of patients who might not be included in our study is small.

Third, prevalence may have been underestimated, as establishing a diagnosis of AF requires a recording to detect the arrhythmia (electrocardiogram, echocardiography). Therefore, many cases of silent AF and some cases of paroxysmal AF may have gone undetected, and patients do not consult for that. Therefore, the prevalence in healthcare data does not adequately reflect the true prevalence in the general population.

Fourth, there is a risk of misclassification bias, because the ICPC-2 K78 is not unique to AF but also includes atrial flutter. However, the prevalence of the latter is substantially lower, and many clinical consequences of both diseases are shared.

Fifth, the changes made by general practitioners to the diagnostic label were detected in the older diagnoses at the beginning of the 2000s, when medical records first began to be computerized. This improvement in the quality of the registry is due to improvements in the knowledge and training of general practitioners in the coding of episodes, as well as to the choice of the exact diagnostic field with no subsequent modifications. In addition, the improved quality of the registry is the result of improvements in information systems that have incorporated ICPC-2 coding dictionaries to guide the professional in the coding process.

Lastly, our study selected the episodes by codes without considering the diagnostic field. Consequently, selection bias may arise in cases where the general practitioner recording the episode modifies the field. For example, 1.09% of the AF codes had a diagnostic label that did not correspond to the episode. On the other hand, a similar proportion (0.9%) of the diagnostic fields of patients without an episode code had been modified and corresponded to AF.

Although validation is probably better for each year that primary care professionals gain experience and better knowledge of their assigned population, given the promising results found, a trend analysis has not been deemed necessary.

## Conclusions

We have estimated the prevalence of atrial fibrillation recorded in the primary care EMR as an approximation of the prevalence in the general population. It is an approximation, given that due to the characteristics of the Spanish National Health System, patients diagnosed with atrial fibrillation in the hospital subsequently go to primary care to continue monitoring their pathology, obtain the prescription for the drugs and carry out the analytical controls (anticoagulation). In addition, they receive help for therapeutic adherence and solving doubts.

Therefore, the atrial fibrillation registries gather the diagnoses made by primary care and those made in the hospital, showing a reasonable estimate of the population prevalence. However, it is impossible to know the population with silent atrial fibrillation, which is not attended to in the healthcare system. The only method to know registered atrial fibrillation and silent atrial fibrillation is to carry out a population screening, which has not been the aim of our study.

The results obtained show that diagnoses of AF in primary care EMR can be used for epidemiological studies.

The estimated prevalence of AF in people over 18 years of age in the Community of Madrid is 2.41%. This percentage is higher in men (2.58%) than in women (2.25%). Prevalence increases progressively with age and does so faster from the age of 60 onwards, reaching 16.95% in people over 80 years of age (19.44% in men and 15.55% in women).

## Data Availability

The datasets generated during the current study are available from the corresponding author on reasonable request.

## Abbreviations

AF: atrial fibrillation
EMR: electronic medical records
CI: confidence interval
ICD-9: International Classification of Diseases, Ninth Revision
ICD-10: International Classification of Diseases, Tenth Revision
ICPC-2: International Classification of Primary Care, 2nd Edition
NPV: negative predictive value
PPV: positive predictive value
